# A new modality of colorectal cancer screening based on chronic disease management

**DOI:** 10.1186/s12876-023-02698-3

**Published:** 2023-03-17

**Authors:** Mo Liu, Shi-Jun Liu, Ming-Jun Chen, Tingting Ning

**Affiliations:** 1 Department of Gastroenterology, Beijing Friendship Hospital, Capital Medical University, National Clinical Research Center for Digestive Diseases, Beijing Digestive Disease Center, Beijing Key Laboratory for Precancerous Lesion of Digestive Diseases, Beijing, 100050 China; 2 Medical Department, Fengtai District You’ anmen Community Health Service Center, Beijing, 100069 China

**Keywords:** Screening modality, Screening participation rate, Colorectal cancer, Chronic disease management, Intervention study

## Abstract

**Background:**

To develop a new modality of colorectal cancer screening based on chronic disease management (CDM) to improve the participation rate of screening, and maximize the benefits of limited resources.

**Methods:**

Patients under CDM were assigned to screening intervention group (SI) and screening control group1 (SC1), residents from natural community were assigned to screening control group2 (SC2). A parallel controlled community intervention study was performed. Only SI would achieve “one-to-one” intervention services. Meanwhile, 200 subjects were selected from each of the three groups for the Knowledge-Attitude-Practice (KAP) questionnaire before and after intervention, named questionnaire intervention group(QI), questionnaire control group1(QC1) and questionnaire control group2(QC2). The outcome of the intervention was evaluated using the difference-in-differences method and multiple regression analysis.

**Results:**

The preliminary screening participation rate was 43.63%(473/1084) in SI, 14.32%(132/922) in SCI, and 5.87%(105/1789) in SC2. The baseline questionnaire showed low knowledge scores in the three questionnaire groups with no statistically significant differences, while attitude scores in QI and QC1 were significantly higher than QC2. The differences between baseline and terminal showed QI increased larger in knowledge and attitude scores than QC1 and QC2, while no difference was detected between QC1 and QC2.

**Conclusion:**

The colorectal cancer screening model based on chronic disease management effectively improved the screening participation rate, and the “one-to-one” intervention and the inherent characteristics of the patient population under CDM were the core elements of the new modality.

**Supplementary Information:**

The online version contains supplementary material available at 10.1186/s12876-023-02698-3.

## Background

### Colorectal cancer screening modalities and their characteristics

Colorectal cancer (CRC) has a high incidence rate in countries worldwide [1, 2]. Studies have shown that precancerous lesions and early cancer can be detected through screening, which can effectively reduce the morbidity and mortality of cancers [3–5].

At present, two modalities of CRC screening are adopted internationally, population-based screening and opportunistic screening [6]. Considering the characteristics of CRC, such as the fact that most patients do not manifest symptoms at an early stage, large-scale screening in the asymptomatic natural population (population-based screening) is the most ideal screening method [7, 8]. However, it is difficult to conduct large-scale population-based screening in China, mainly due to issues such as poor compliance with population-based screening [9], the large population base, high financial costs, and the shortage of health and human resources [10]. Although opportunistic screening is a simple and economical screening method [11, 12] and can achieve a high participation rate, it is usually conducted at a gastroenterology clinic in secondary and tertiary general hospitals and covers a relatively small population. Thus, compared to population-based screening, opportunistic screening has limited social benefits.

Therefore, it is imperative to develop a new screening modality with the advantages of both modalities, which not only retains a high screening participation rate and has an economical and simple operation but also covers a larger population, to maximize the benefits of limited resources and meet the needs of the current situation in China and other developing countries.

### Colorectal cancer screening modality based on chronic disease management

China has a large population with chronic noninfectious diseases [13]. In recent years, with continuous improvement of the community health service system, chronic disease management (CDM) has gradually undergone standardization, and the population under CDM is growing. The CDM team at community health service centers establishes chronic disease files for patients; develops systems for regular examinations and follow-up; organizes regular activities such as health seminars to increase the awareness, knowledge, and operational skills related to disease management and healthy life styles; and establishes a beneficial and stable doctor-patient relationship [14, 15].

Compared to secondary and tertiary general hospitals, community health service centers cover a broader range of patients under CDM, most of whom meet the age requirements for CRC screening (40–74 years). These patients have a high degree of health awareness and compliance, which provides a basis for the screening. The regular follow-up evaluations of the CDM system ensure the accessibility of intervention, while their well-developed health record system facilitates follow-up evaluations after screening.

Therefore, by utilizing the inherent characteristics of the population under CDM and the features of the management system, the exploration of a new modality of CRC screening based on CDM will improve the participation rate of CRC screening, reduce screening costs, and maximize the benefits of limited resources.

## Materials and methods

### Population

#### The screening groups

The You’anmen Community in Beijing, China, was selected as the study site in March 2014. Patients who had undergone visits within 3 months and had medical records in the CDM system at the health service centers of You’anmen Community were enrolled into screening intervention group (SI, center 1, 1084 subjects) and screening control group1 (SC1, center 2, 922 subjects). Residents from one of You’anmen sub-communities registered at the neighborhood committee were enrolled into screening control group2 (SC2, 1789 subjects). CRC screening was performed in those groups within the same time period.

### The Questionnaire Groups

A total of 600 subjects were selected from these three screening groups (200 subjects selected respectively from each group). The sampling method was based on the chronological order of the visits of the subjects in SI and SC1. These first 200 subjects who visited the center 1 were enrolled as the questionnaire intervention group (QI). Likewise, the first 200 subjects who visited the center 2 were enrolled as the questionnaire control group1 (QC1). The rest 200 subjects in questionnaire control group2 (QC2) were randomly selected from the SC2.

Inclusion criteria: subjects aged 40–74 years.

Exclusion criteria: (1) subjects who had been definitely diagnosed with CRC; (2) subjects who had severe heart, brain, lung, liver, or kidney dysfunction or mental illness.

### Study Design

The community intervention study was adopted with a parallel control. There was a CRC screening program holding at the You’anmen Community during our study. Anyone could participate in this program for free. All these three screening groups could realize the program by educational materials placing at the public area, such as the health service centers and communities. Educational materials included display panels, banners, and pamphlets. However, only the SI would achieve the “one-to-one” intervention services which are recommending CRC screening and distributing educational pamphlets by physicians and nurses at the time of outpatient follow-up or telephone follow-up.

**Fig. 1:**
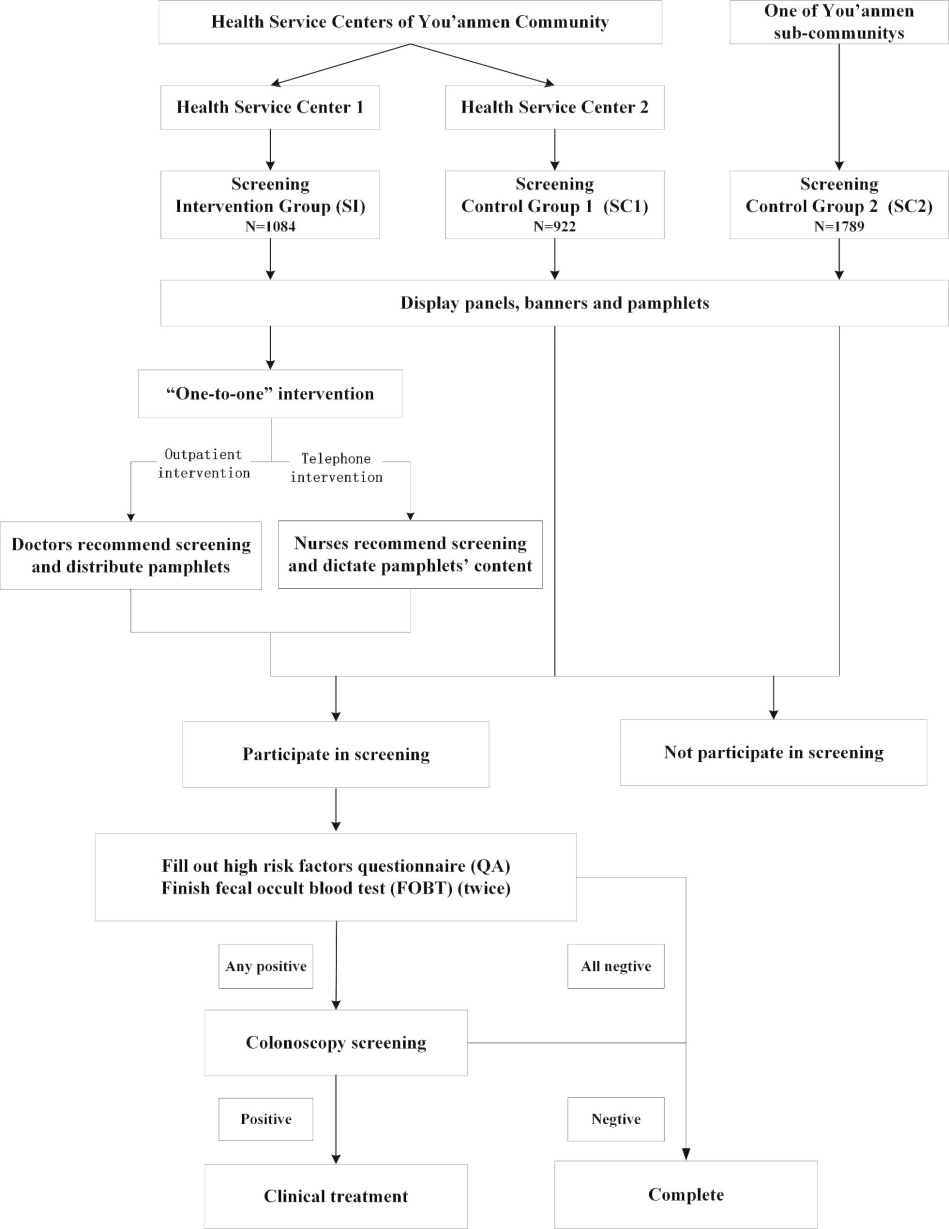
Screening and Intervention Programs

Those who wished to participate in the screening completed the high risk factors questionnaire (QA) in combination with the fecal occult blood test (FOBT), according to the “Technical Program for Cancer Screening and Early Diagnosis and Treatment in China”. The healthcare personnel issued a QA and 2 FOBT kits to participants, and instructed participants to complete the QA and collected them back once done. In the next two weeks, the participants collected their stool samples twice with the issued FOBT kits, and returned them to hospital respectively. A positive result from either of the two tests was considered a positive FOBT. Subjects with any positive results on the QA or FOBT during the screening were classified into the high-risk population and were recommended to be checked by colonoscopy (Fig. 1).

**Fig. 2:**
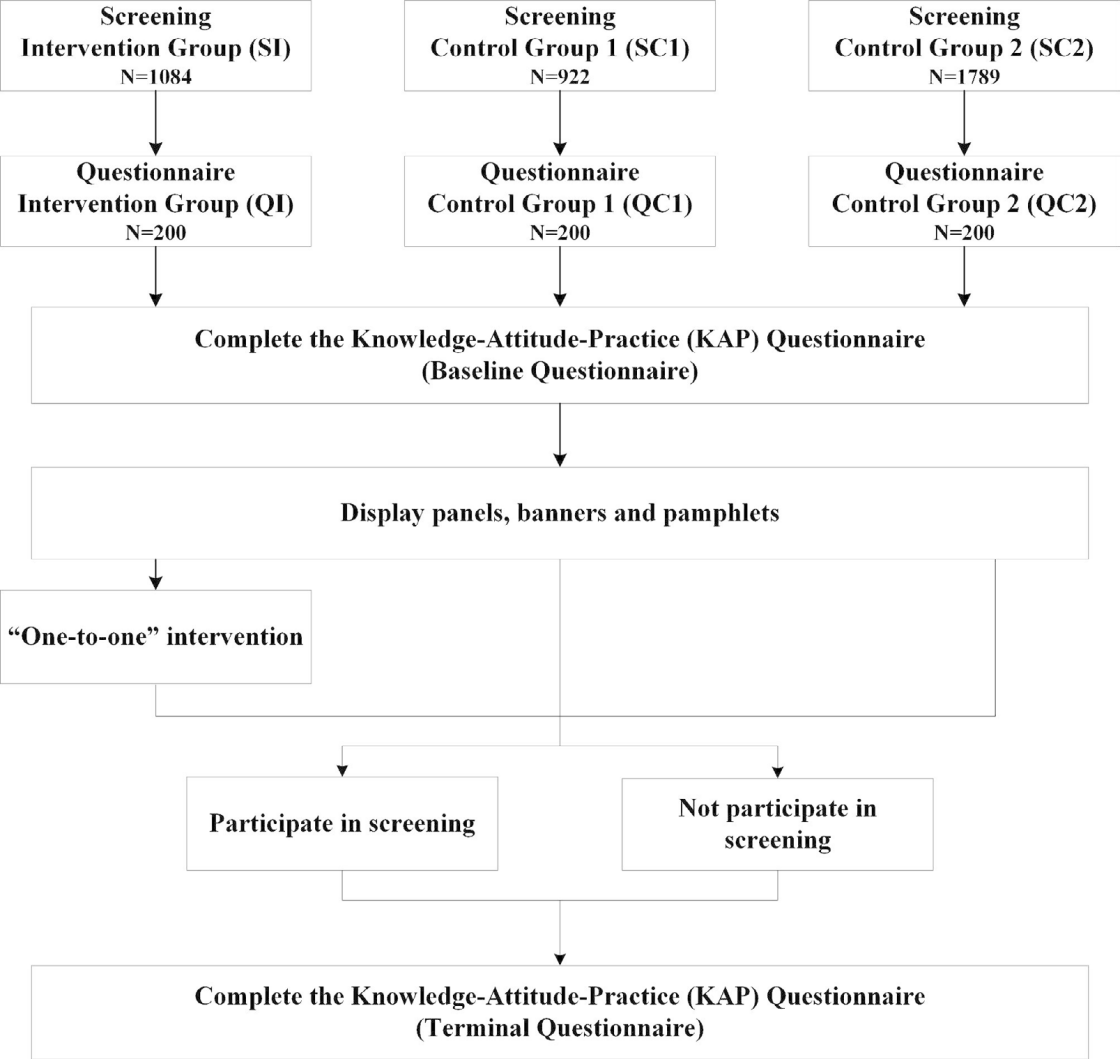
Screening and Intervention Programs with Questionnaire

Meanwhile, 600 subjects from these three questionnaire groups completed the Knowledge-Attitude-Practice (KAP) questionnaire before and after the intervention. The survey was conducted by trained college students and community healthcare personnel. The baseline questionnaire was conducted in March 2014, and the questionnaire content was described in a previously published study [16]. The terminal questionnaire survey was conducted in December 2014, and its content was consistent with that of the baseline questionnaire (Fig. 2).

### Outcome measurement

The main indicators for evaluation of the intervention effects in this study were as follows: (1) participation rate of CRC screening and (2) differences of knowledge and attitude scores between baseline and terminal. The knowledge items involved a total of ten questions, including seven single-choice questions and three multiple-choice questions, for a total of 24 points. The attitude items were measured using the Likert five-point method, with answers ranging from “completely agree” to “completely disagree”, with a score of 5 to 1 points based on a positive statement. In contrast, for a negative statement, the grades ranged from 1 to 5. A total of nine questions were asked, with a total of 65 points.

### Statistical analysis

Binary non-conditional logistic regression analysis was used to compare the differences of participation rates among these three groups. Then, t test, ANOVA or rank-sum test were used to compare the differences of knowledge and attitude scores among these three groups before and after the intervention. The difference-in-differences model of independent pooled cross-sectional data was used to evaluate the effects of interventions regarding the knowledge and attitude of the subjects, which involved the establishment of multiple linear regression model that included knowledge and attitude scores as the dependent variables, group, time points, interaction of time points and group, age groups, and sex as the independent variables.

The difference-in-differences model of independent pooled cross-sectional data was calculated as follows:

**Y**_**it**_**=b**_**0**_ **+ b**_**1**_·**T**_**it**_**+b**_**2**_·**A**_**it**_**+b**_**3**_·**T**_**it**_·**A**_**it**_**+e**_**it**_ where Y represents the dependent variable, T and A represent the time points and groups respectively, T·A represents the interaction of time points and group, e indicates the residual error, i = 0 and 1 represent the control and intervention groups respectively, and t = 0 and 1 indicate the baseline and terminal survey. All statistical analyses were performed using STATA version 12.0.

## Results

### Screening rate

A total of 1084, 922, and 1789 subjects were enrolled in SI, SC1 and SC2, respectively. The participation rates in the screening were 43.63% (473/1084) in SI, 14.32% (132/922) in SC1, and 5.87% (105/1789) in SC2.

To exclude interference from factors such as age and sex and determine the difference in the screening participation rates among these three groups, the group, age, and sex were included in the logistic regression model. A regression model was obtained for the population represented by these three groups through screening of the variables via the stepwise regression method, using SC2 as the control. The results showed a statistically significant difference when the SI and SC1 compared with SC2, both with P < 0.001. The participation rate of the screening intervention group was 39.72% higher than that of SC2, while the participation rate of SC1 was 10.85% higher than that of SC2 (Table 1).

**Table 1:** Screening Participation among Three Screening Groups

Variable	Odds Ratio (95% Conf. Interval)	dy/dx	P
**Screening Control Group 2 (SC2)**	Reference	**-**	-
**Screening Intervention Group (SI)**	11.72(9.29,14.79)	**0.397**	**< 0.001**
**Screening Control Group 1 (SC1)**	2.34(1.78,3.07)	**0.109**	**< 0.001**
Age	1.02(1.01,1.03)	0.003	0.001
Gender	0.58(0.48,0.71)	-0.066	< 0.001
Constant	0.02(0.01,0.05)		< 0.001

A regression model was used to analyze the data of the SI and SC1 through screening of the variables via the stepwise regression method, with the group, age, sex, marital status, educational level, occupation and average household monthly income per capita included in the logistic regression model. The results showed a statistically significant difference in the group identity with P < 0.001. The participation rate of the SI was 29.01% higher than that of SC1 (Table 2).

**Table 2:** Screening Participation between Screening Intervention Group and Screening Control Group 1

Variable	Odds Ratio (95%Conf.Interval)	dy/dx	P
**Group**	4.66(3.71,5.85)	0.290	**< 0.001**
Age	1.01(0.99,1.03)	0.002	0.333
Gender	0.70(0.56,0.87)	-0.067	0.001
Marital Status	1.28(0.79,2.06)	0.047	0.314
Education 1	2.10(1.33,3.31)	0.126	0.001
Education 2	2.25(1.31,3.86)	0.162	0.003
Occupation	1.36(0.96,1.92)	0.055	0.081
Income/Capita/Month	1.00(1.00,1.00)	-0.00001	0.366
Constant	0.05(0.02,0.17)		< 0.001

### Changes in knowledge-attitude-practice scores

In the baseline survey, 200 copies of the questionnaire were distributed in each of three questionnaire groups, and 193 (96.5%), 189 (94.5%), and 188 (94.0%) valid copies were returned from the QI, QC1, and QC2, respectively. No significant differences were found in the basic information including sex, age, marital status, educational level, occupation and average monthly household income per capita (*P* > 0.05) (Table S1). In the terminal survey, the subjects were followed up according to the subject list from the baseline survey. A total of 412 subjects were surveyed, including 149 (77.2%) subjects from QI, 139 subjects (73.5%) from QC1, and 124 subjects (66.0%) from QC2. No statistically significant differences were found in the basic information of the subjects in the terminal survey among these three groups (*P* > 0.05) (Table S2). In addition, no statistically significant differences in the basic information of the subjects between the baseline and terminal survey in each of these three groups (Table 3).

**Table 3:** Comparisons of Basic Information between Baseline and Terminal Survey in Three Questionnaire Groups

	Questionnaire Intervention Group				Questionnaire Control Group 1					Questionnaire Control Group 2					
	**Baseline**	**Terminal**	**χ2**	**P**	**Baseline**	**Terminal**		**χ2**	**P**	**Baseline**	**Terminal**		**χ2**	**P**	
**Gender**			0.35	0.557			0.36		0.547				0.33	0.564	
Male	72(37.3%)	51(34.2%)			70(37.0%)	47(33.8%)				82(43.6%)		50(40.3%)			
Female	121(62.7%)	98(65.8%)			119(63.0%)	92(66.2%)				106(56.4%)		74(59.7%)			
**Age**			0.85	0.837			0.04		0.998				1.93	0.588	
40–49	10(5.2%)	9(6.0%)			9(4.8%)	7(5.0%)				21(11.2%)		9(7.3%)			
50–59	87(45.1%)	71(47.7%)			70(37.0%)	51(36.7%)				73(38.8%)		52(41.9%)			
60–69	79(40.9%)	54(36.2%)			90(47.6%)	67(48.2%)				78(41.5%)		55(44.4%)			
70–74	17(8.8%)	15(10.1%)			20(10.6%)	14(10.1%)				16(8.5%)		8(6.5%)			
**Marital status**			0.04	0.851			0.12		0.729				0.15	0.700	
Married	176(91.2%)	14(9.4%)			172(91.0%)	11(7.9%)				177(94.1%)		6(4.8%)			
Unmarried	17(8.8%)	135(90.6%)			17(9.0%)	128(92.1%)				11(5.9%)		118(95.2%)			
**Education**			0.001	0.999			0.29		0.866				1.59	0.456	
Illiterate/ Primary School	9(4.7%)	7(4.7%)			15(7.9%)	12(8.6%)				17(9.0%)		7(5.6%)			
Middle School / High School	157(81.3%)	121(81.2%)			159(84.1%)	118(84.9%)				149(79.3%)		99(79.8%)			
Junior College and above	27(14.0%)	21(14.1%)			15(7.9%)	9(6.5%)				22(11.7%)		18(14.5%)			
**Occupation**			0.17	0.677			0.02		0.881				0.12	0.729	
Non-working	170(88.1%)	20(13.4%)			169(89.4%)	14(10.1%)				156(83.0%)		23(18.5%)			
In-service	23(11.9%)	129(86.6%)			20(10.6%)	125(89.9%)				32(17.0%)		101(81.5%)			
**Income/ capita /month**			0.23	0.978			1.91		0.595				1.21	0.750	
Below 2000	12(6.2%)	10(6.7%)			22(11.6%)	12(8.6%)				15(8.0%)		7(5.6%)			
2000~	151(78.2%)	115(77.2%)			134(70.9%)	102(73.4%)				145(77.1%)		94(75.8%)			
4000~	27(14.0%)	22(14.8%)			27(14.3%)	23(16.5%)				21(11.2%)		17(13.7%)			
6000 and more	3(1.6%)	2(1.3%)			6(3.2%)	2(1.4%)				7(3.7%)		6(4.8%)			

**Fig. 3:**
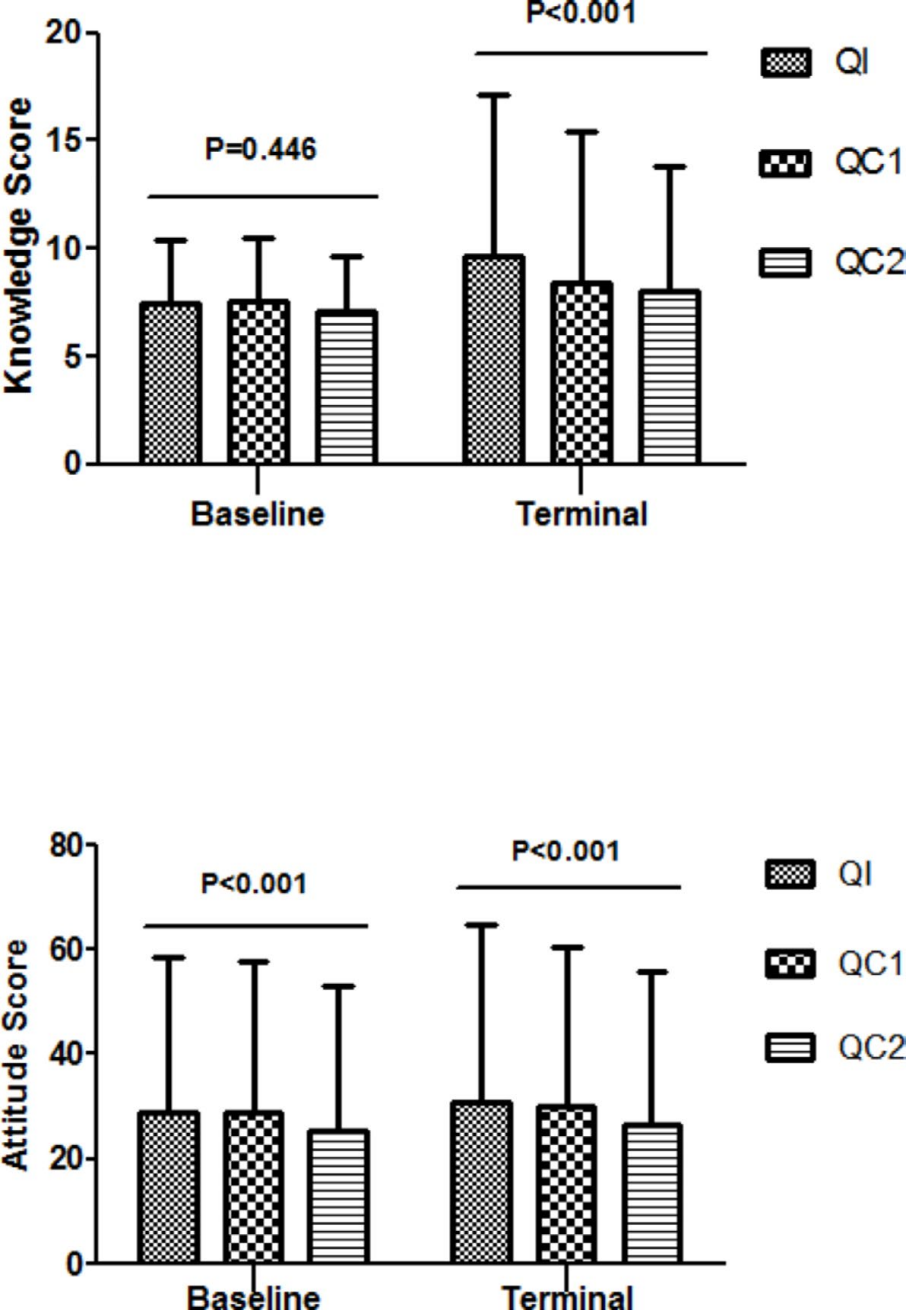
Knowledge and Attitude Scores Comparisons of Baseline and Terminal Survey among Three Questionnaire Groups

The statistical analysis results showed that the baseline knowledge scores of the QI, QC1, and QC2 were 9.58, 9.67, and 8.90, respectively, and the baseline attitude scores were 49.89, 49.20, and 45.15, respectively. No significant difference was observed in the knowledge scores among these three groups (*P* = 0.446), while the attitude scores exhibited statistically significant differences (*P* < 0.001, no difference was found between the QI and QC1). The terminal knowledge scores of these three groups were 14.99, 13.40, and 12.16, respectively, and the terminal attitude scores were 55.04, 51.68, and 47.20, respectively. These three groups showed statistically significant differences in the terminal knowledge and attitude scores (*P* < 0.001), with the highest scores in QI, followed by QC1, and the lowest scores were found in QC2 (Fig. 3). Compared to the baseline scores, all groups exhibited increases in terminal knowledge and attitude scores, with statistically significant differences (Table S3).

**Fig. 4:**
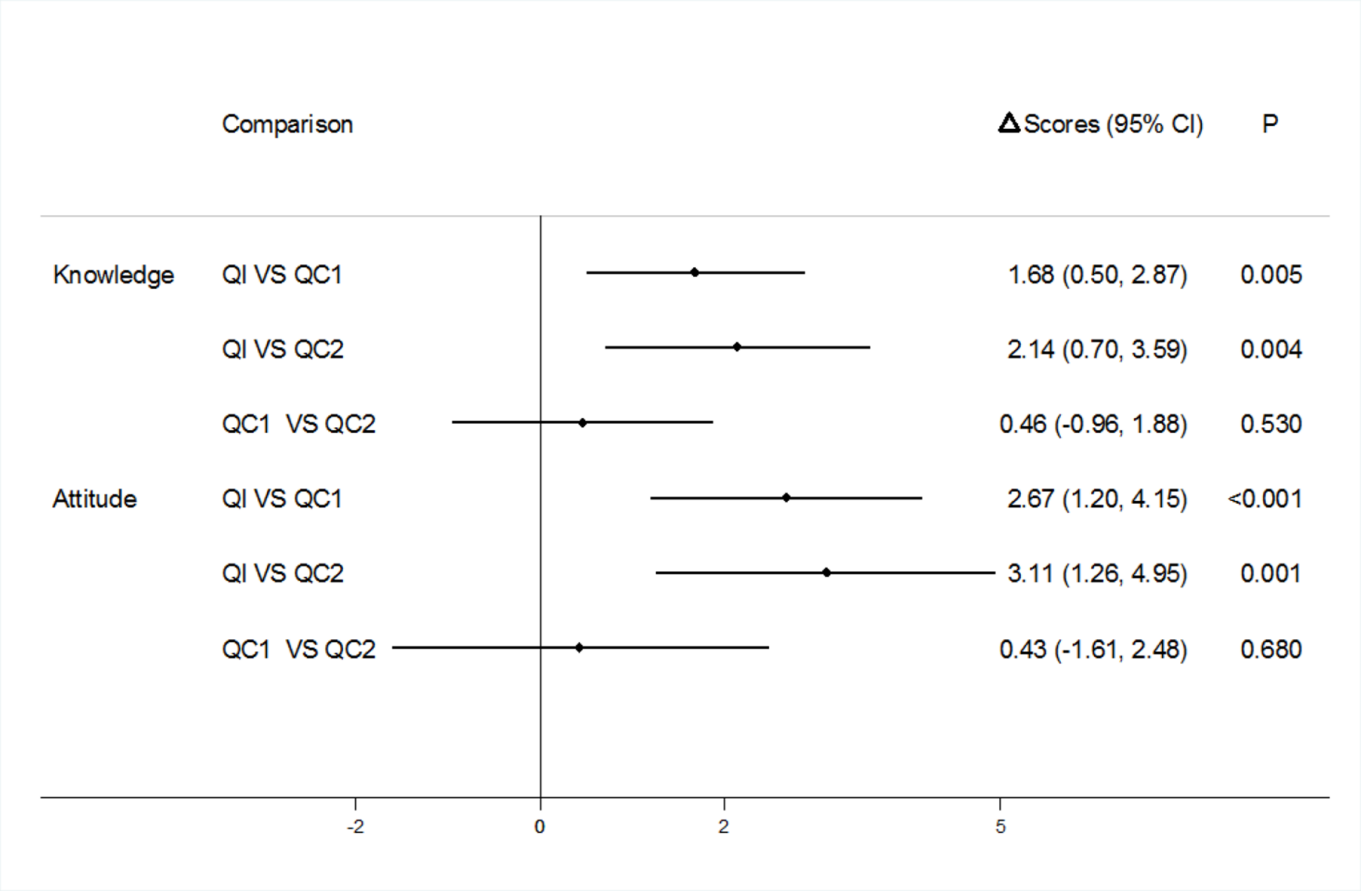
Differences of Knowledge and Attitude Scores between Baseline and Terminal Survey among Three Questionnaire Groups

To control for the effect of baseline and confounding variables on the changes in knowledge and attitude scores and to understand the net effect of the intervention, the data were further analyzed using the difference-in-differences method. The results showed the greatest increases of knowledge and attitude scores were in QI. The increase of the knowledge score in QI was 1.68 points higher than that in QC1 (*P* = 0.005) and 2.14 points higher than that in QC2 (*P* = 0.004), while the increase of the attitude score in QI was 2.67 points higher than that in QC1 (*P* < 0.001) and 3.11 points higher than that in QC2 (*P* = 0.001). No significant differences were observed in the increase of the knowledge and attitude scores between QC1 and QC2 (Fig. 4).

## Discussion

### Colorectal cancer screening based on chronic disease management effectively improved the screening participation rate

The screening participation rate is the most direct indicator for evaluation of the effect of an intervention. In this study, the screening participation rate was 43.63% in SI, 14.32% in SC1, and 5.87% in SC2. Multivariate logistic regression analysis showed the highest screening participation rate in the population under CDM who received the “one-to-one” intervention (SI), which was followed by the participation rate of the population under CDM who did not receive the “one-to-one” intervention (SC1). The lowest screening participation rate was found in the natural population who did not receive the “one-to-one” intervention (SC2). This result demonstrated that CRC screening based on CDM effectively improved the screening participation rate.

**“One-to-one” intervention and the inherent characteristics of the population under chronic disease management were the core elements of the chronic disease management modality**.

The analysis results showed the highest increases of knowledge and attitude scores were all in the population under CDM who received the “one-to-one” intervention (QI). In addition, although the subjects in QC1 and QI all belonged to the population under CDM and had higher baseline attitude scores in the screening, the increases of the knowledge and attitude scores in QC1 were smaller than those in QI, suggesting that the “one-to-one” intervention improved the knowledge and attitude scores of the population under CDM. Other articles also indicated that intervention from a physician is very important for determining whether a patient is screened for CRC, especially for individuals who have access to and make use of healthcare services [17, 18].

Although no significant differences were observed in the increases of knowledge and attitude scores between QC1 and QC2, the baseline and terminal attitude scores in QC1 were higher than those in QC2, indicating that the population under CDM had a better understanding of the screening, might have paid more attention to their health due to their chronic diseases, and were more willing to participate in a variety of activities conducive to their health. Therefore, more “one-to-one” interventions could more effectively improve the screening participation rate.

**Improving the intervention capability of healthcare personnel and the quality of educational materials specifically based on the characteristics of the population under chronic disease management are indispensable**.

This study fully showed the critical role of healthcare personnel in the education of population under CDM. Therefore, it is necessary to provide education training to healthcare personnel and to stress their obligation to improve the screening participate rate. When providing intervention to the population under CDM, healthcare personnel should reduce the use of professional terminology and avoid sensitive topics such as mortality. More importantly, healthcare personnel should focus more on explaining the value of the screening and praising target population’s existing high health awareness. Therefore, they will have a stronger desire to participate in the screening and complete all screening procedures.

Meanwhile, because the population under CDM is mostly middle-aged or elderly and has decreased literacy levels, the educational materials for screening should be easy to understand, i.e. with more figures, illustrations, shorter descriptions. High-quality posters or manuals will also reduce the workload of healthcare personnel, facilitate a deeper understanding of related knowledge by the target population, and further improve screening participation rate.

### Potential implications for clinical practice needs further research

The modality of this study is not a new clinical screening tool, but a new screening management modality. However, it would achieve better effect for improving the screening rate and efficiency along with clinical screening tools, such as some bio-makers and colonoscopy, et al., because it based on the specific population’s high compliance, smooth doctor-patient relationship and easy to educate. In addition, it can also help the established clinical screening tools play a bigger role in routine clinical practice.

Furthermore, the potential implications of the new modality in the surveillance also provide profound significance. Generally, it is efficient to perform a regular follow-up for the population under CDM, as they have continuous health records and stable doctor-patient relationship. In this circumstance, it would be a better choice if we take full advantages from the above model to establish a long-term surveillance to provide evidence for screening strategy, i.e. screening frequency and clinical tools, etc.

## Limitation

This study was conducted in Beijing, the capital of China, where the chronic disease management was at a higher level due to the developed economics and higher level of education. Therefore, colorectal cancer screening based on chronic disease management needs to be further validated among other regions.

In addition, CRC screening based on CDM does not require excessive human and material resources, which reduces the cost and time. Further study could focus on the economic evaluation of the cost-benefit of screening which will facilitate validation of the feasibility and effectiveness of this screening modality.

## Conclusion

Population under CDM is widely found in community groups, and these individuals usually have a higher screening rate due to their increased compliance and more opportunities for “one-to-one” intervention. Therefore, a long-term effective CRC screening mechanism based on CDM should be established in China and other developing countries, in which the population under CDM receives health interventions from community healthcare personnel according to the KAP principles at the time of regular follow-up evaluations. This will improve the screening efficiency and better utilize limited resources.

## Electronic supplementary material

Below is the link to the electronic supplementary material.

Supplementary Material 1. Table S1. Comparisons of Basic Information of the Baseline Survey among Three Questionnaire Groups. Table S2. Comparisons of Basic Information of the Terminal Survey among Three Questionnaire Groups. Table S3. Knowledge and Attitude Scores Comparisons between Baseline and Terminal Survey in Three Questionnaire Groups.

## Data Availability

The datasets used and/or analyzed during the current study available from the corresponding author on reasonable request.
