# Supplementary material for: A new modality of colorectal cancer screening based on chronic disease management
Source: BMC Gastroenterol. 2023 Mar 17;23:78. doi: 10.1186/s12876-023-02698-3 (PMC10024352; doi:10.1186/s12876-023-02698-3)
Supplement: Supplementary file 1 — Supplementary Material 1. Table S1. Comparisons of Basic Information of the Baseline Survey among Three Questionnaire Groups. Table S2. Comparisons of Basic Information of the Terminal Survey among Three Questionnaire Groups. Table S3. Knowledge and Attitude Scores Comparisons between Baseline and Terminal Survey in Three Questionnaire Groups. [file 12876_2023_2698_MOESM1_ESM.doc]

**Table S1 Comparisons of Basic Information of the Baseline Survey among Three Questionnaire Groups**

|  | **Questionnaire**  **Intervention Group**  **(QI)** | **Questionnaire Control Group 1**  **(QC1)** | **Questionnaire Control Group 2**  **(QC2)** | **χ2** | **P** |
| --- | --- | --- | --- | --- | --- |
| **Gender** |  |  |  | 2.20 | 0.333 |
| Male | 72(37.3%) | 70(37.0%) | 82(43.6%) |  |  |
| Female | 121(62.7%) | 119(63.0%) | 106(56.4%) |  |  |
| **Age** |  |  |  | 10.32 | 0.112 |
| 40-49 | 10(5.2%) | 9(4.8%) | 21(11.2%) |  |  |
| 50-59 | 87(45.1%) | 70(37.0%) | 73(38.8%) |  |  |
| 60-69 | 79(40.9%) | 90(47.6%) | 78(41.5%) |  |  |
| 70-74 | 17(8.8%) | 20(10.6%) | 16(8.5%) |  |  |
| **Marital status** |  |  |  | 1.62 | 0.446 |
| Married | 176(91.2%) | 172(91.0%) | 177(94.1%) |  |  |
| Unmarried | 17(8.8%) | 17(9.0%) | 11(5.9%) |  |  |
| **Education** |  |  |  |  |  |
| Illiterate/ Primary School | 9(4.7%) | 15(7.9%) | 17(9.0%) | 6.21 | 0.184 |
| Middle School / High School | 157(81.3%) | 159(84.1%) | 149(79.3%) |  |  |
| Junior College and above | 27(14.0%) | 15(7.9%) | 22(11.7%) |  |  |
| **Occupation** |  |  |  | 3.81 | 0.149 |
| Non-working | 170(88.1%) | 169(89.4%) | 156(83.0%) |  |  |
| In-service | 23(11.9%) | 20(10.6%) | 32(17.0%) |  |  |
| **Income/ capita /month** |  |  |  | 6.76 | 0.344 |
| Below 2000 | 12(6.2%) | 22(11.6%) | 15(8.0%) |  |  |
| 2000~ | 151(78.2%) | 134(70.9%) | 145(77.1%) |  |  |
| 4000~ | 27(14.0%) | 27(14.3%) | 21(11.2%) |  |  |
| 6000 and more | 3(1.6%) | 6(3.2%) | 7(3.7%) |  |  |

**Table S2 Comparisons of Basic Information of the Terminal Survey among Three Questionnaire Groups**

|  | **Questionnaire**  **Intervention Group**  **(QI)** | **Questionnaire Control Group 1**  **(QC1)** | **Questionnaire Control Group 2**  **(QC2)** | **χ2** | **P** |
| --- | --- | --- | --- | --- | --- |
| **Gender** |  |  |  | 1.50 | 0.473 |
| Male | 51(34.2%) | 47(33.8%) | 50(40.3%) |  |  |
| Female | 98(65.8%) | 92(66.2%) | 74(59.7%) |  |  |
| **Age** |  |  |  | 6.37 | 0.384 |
| 40-49 | 9(6.0%) | 7(5.0%) | 9(7.3%) |  |  |
| 50-59 | 71(47.7%) | 51(36.7%) | 52(41.9%) |  |  |
| 60-69 | 54(36.2%) | 67(48.2%) | 55(44.4%) |  |  |
| 70-74 | 15(10.1%) | 14(10.1%) | 8(6.5%) |  |  |
| **Marital status** |  |  |  | 2.07 | 0.356 |
| Married | 14(9.4%) | 11(7.9%) | 6(4.8%) |  |  |
| Unmarried | 135(90.6%) | 128(92.1%) | 118(95.2%) |  |  |
| **Education** |  |  |  | 6.95 | 0.139 |
| Illiterate/ Primary School | 7(4.7%) | 12(8.6%) | 7(5.6%) |  |  |
| Middle School / High School | 121(81.2%) | 118(84.9%) | 99(79.8%) |  |  |
| Junior College and above | 21(14.1%) | 9(6.5%) | 18(14.5%) |  |  |
| **Occupation** |  |  |  | 3.98 | 0.136 |
| Non-working | 20(13.4%) | 14(10.1%) | 23(18.5%) |  |  |
| In-service | 129(86.6%) | 125(89.9%) | 101(81.5%) |  |  |
| **Income/ capita /month** |  |  |  | 4.92 | 0.556 |
| Below 2000 | 10(6.7%) | 12(8.6%) | 7(5.6%) |  |  |
| 2000~ | 115(77.2%) | 102(73.4%) | 94(75.8%) |  |  |
| 4000~ | 22(14.8%) | 23(16.5%) | 17(13.7%) |  |  |
| 6000 and more | 2(1.3%) | 2(1.4%) | 6(4.8%) |  |  |

**Table S3 Knowledge and Attitude Scores Comparisons between Baseline and Terminal Survey in Three Questionnaire Groups**

|  | **Knowledge Score (KS)** | | | |  | **Attitude Score (AS)** | | | |
| --- | --- | --- | --- | --- | --- | --- | --- | --- | --- |
|  | **Baseline** | **Terminal** | **t(t’)** | **P** |  | **Baseline** | **Terminal** | **t(t’)** | **P** |
| **Questionnaire Intervention Group (QI)** | 9.58±5.40 | 14.99±4.41 | -9.48 | <0.001 |  | 49.89±7.46 | 55.04±6.78 | -6.24 | <0.001 |
| **Questionnaire Control Group 1**  **(QC1)** | 9.67±5.44 | 13.40±4.37 | -6.30 | <0.001 |  | 49.20±8.29 | 51.68±8.31 | -2.49 | 0.013 |
| **Questionnaire Control Group 2**  **(QC2)** | 8.90±5.27 | 12.16±3.95 | -5.52 | <0.001 |  | 45.15±5.78 | 47.20±5.74 | -2.80 | 0.006 |
